# Hurricane Irma Linked to Coral Skeletal Density Shifts on the Florida Keys Reef Tract

**DOI:** 10.1093/icb/icae128

**Published:** 2024-08-05

**Authors:** Griffith Aliyah, Sanchez Gomez Jose, Castillo Karl

**Affiliations:** Department of Earth Marine and Environmental Science, University of North Carolina Chapel Hill, Chapel Hill, NC 27514, USA; Department of Statistics, University of California Riverside, Riverside, CA 92521, USA; Department of Earth Marine and Environmental Science, University of North Carolina Chapel Hill, Chapel Hill, NC 27514, USA

## Abstract

Coral reefs are at risk due to various global and local anthropogenic stressors that impact the health of reef ecosystems worldwide. The most recent climate models predict that climate change will increase the frequency and intensity of tropical storms. This increased storm occurrence and strength will likely compromise coral reef structures and habitats for reef-dwelling organisms, including across the Florida Keys Reef Tract (FKRT), the most extensive tropical reef system along the US coast. While several recent studies reveal the chronic impacts of tropical storms on corals, relatively little is known about the effects of major storm events on coral growth and how these effects vary over spatiotemporal scales. Here, I characterize the skeletal growth of two common Caribbean reef-building coral species, *Siderastrea siderea* and *Pseudodiploria strigosa*, before and after Hurricane Irma to investigate the storm’s impact on coral skeletal growth on inner and outer reefs of the FKRT. Coral cores were extracted from both species at four inner and four outer reef sites in May 2015, before Hurricane Irma struck the Florida Keys in September 2017. Subsequently, 33 micro-cores were collected in May 2019, two years after the storm traversed our previously cored coral colonies. A three-way ANOVA model with storm, species, and reef location as the three factors was used to assess the impact of the storm on each of three growth parameters: skeletal density, linear extension, and calcification rates. Results reveal no difference in the coral annual skeletal growth parameters pre- and post-Hurricane Irma, although previously quantified differences in these growth parameters across species and location were observed. However, analysis of the “yearly” change in annual skeletal growth parameters showed significant differences in skeletal density across groups before and after Hurricane Irma, but not for linear extension and calcification rates. Our findings improve an understanding of the impacts of tropical storms on coral skeletal growth and offer new insights into how we can employ corals’ innate growth capacities to help conserve coral reefs under climate change.

## Introduction

Reef-building corals worldwide are under significant threats from various global and local stressors. Global stressors include climate change-induced ocean warming and acidification causing bleaching and impacting coral infrastructure (Hughes 1994; Anthony et al. 2011; Hughes and Grottoli 2013). The Florida Keys Reef Tract (FKRT) is a chain of islands off the coast of South Florida, stretching almost 350 miles from the Dry Tortugas in the south to the St. Lucie Inlet in the north. On the FKRT, threats to corals from climate change are compounded by an extensive list of local stressors (Carpenter et al. 2008; Kuffner et al. 2015). The FKRT is known for its widespread coastal development (Cunning et al. 2019) and recurrent disease outbreaks (Walker 2018; Walton et al. 2018; Rippe et al. 2019), all of which have had devastating effects on the reef ecosystem health. Here ecosystem health is defined as the reef effectively maintaining its overall condition and ecological functions and sustaining vital ecosystem services for coastal communities. As the health of corals on the FKRT continues to decline, it is essential to assess all of the factors contributing to the overall decline in coral abundance, species diversity, and physical condition shifts across the reef tract. Unlike other local factors, tropical storms and hurricanes are known to introduce environmental changes but also exacerbate physical damage to coral reefs (Lirman and Fong 1997; Hughes and Connell 1999; Gardner et al. 2005). Hurricanes impact corals directly and cause shifts in environmental characteristics resulting in further stress to the reef system (Edmunds et al. 2019).

### Hurricane impacts on the environment

Hurricanes are known to cause physical damage to corals through increased wave height and storm surge (Rogers et al. 1991). The acute disturbance generated by hurricanes precipitates further damage, influencing environmental characteristic shifts such as decreased light availability and increased sedimentation (Edmunds et al. 2019), deterioration of previously sustained coral coverage (Gardner et al. 2005), and temperature fluxes (Wilkinson and Souter 2008). As environmental conditions shift, pre-existing stressors, such as coral disease, can be induced. For example, after Hurricane Mitch, corals across the greater Caribbean had compounded impacts of bleaching and coral disease along their forereefs. The Belize Barrier Reef exhibited the highest coral disease prevalence, though it was not directly hit by the storm (Kramer et al. 2000). Recent studies have modeled Hurricane Irma’s impact on the FKRT, showcasing an accelerated spread of Stony Coral Tissue Loss Disease (SCTLD) by almost 30 days (Dobbelaere et al. 2024).

Hurricanes traversing coral reefs induce a cooling effect on the sea surface while gaining strength (Kramer et al. 2000; Trenberth et al. 2018). During the 2005 season, Hurricane Dennis, Katrina, Rita, and Wilma caused a 2–5°C drop in seawater temperature across the FKRT (Wilkinson and Souter 2008). These temperature fluctuations, known to hinder coral growth and health, are exacerbated by reduced light availability caused by storms such as Hurricane Irma and Maria, leading to decreased coral photosynthetic activity and overall growth (Houlbrèque et al. 2003; Edmunds et al. 2019). This can cause coral percent cover to decline in the years following storm introduction. Through a multidecadal analysis, Gardner et al. (2005) found that since the 1980s, storms can still have significant localized, immediate impacts on coral cover. Based on the typical interval of natural recurrence for hurricanes in regions prone to hurricane activity, no evidence was found of coral recovery post-hurricane impact.

### Changing hurricane characteristics

With hurricane damage detrimentally affecting reefs in multiple ways, the frequency and size of hurricanes have become an even more significant concern. The frequency of hurricane development varies across the globe but is now expected to become larger and more forceful (Knutson et al. 2010), especially in the Florida Keys. Large, strong, and/or a combination of large and strong storms are predicted to strike the FKRT every 2.9–5.7 years (Puotinen et al. 2020). This would only lead to higher stress on the reef ecosystem and potential decreases in overall coral health. With models showing predicted increases in hurricanes, previous dampening effects of many short-term impacts, such as local sea temperature, will be altered because the loss of coral is positively related to hurricane intensity (Gardner et al. 2005).

### Physical damage to coral reefs

Coral skeletal growth parameters are not commonly coupled with hurricanes and most studies assess elements of coral cover loss or physical destruction influences such as fragmentation. Hernández-Delgado et al. (2014), however, found a very fast linear extension for *Acropora cervicornis* in Puerto Rico, a region known for its consistent exposure to hurricanes and sedimentation, after Hurricane Irene. This surprisingly fast growth was nonetheless seen as a noteworthy observation. However, it raised cautious concerns about the potential for corals to possess lower energy reserves and a reduced likelihood of recovery and resilience against future disturbances.

Hurricanes are increasingly having a greater impact, and in 2017, Hurricane Irma made its way through the FKRT, resulting in widespread consequences. Irma arrived at the FKRT on September 10th, 2017, as a Category 3 storm. During its course, it reached Category 5 and maintained winds of 185 mph or higher for 37 h (Cangialosi et al. 2021). The total cost of Irma-related damage was $50 billion, and was ranked the fifth most expensive hurricane to hit the contiguous United States (Torrey and Chaney 2018). As large and more forceful storms occur with more frequency worldwide, this increases the chances of damage along the Florida Keys. The FKRT is known to have serious reductions in coral growth and health with 90% loss due to the combination of disease, storms, and high sea surface temperatures (Soper et al. 2022). Nevertheless, it has sustained some coral reefs despite experiencing high losses from disease, bleaching, and physical destruction. Even so, a recent study found that corals on the FKRT sustained their extension and calcification but not their skeletal density (Rippe et al. 2018). As the frequency and intensity of hurricanes continue to increase along the FKRT, a region already experiencing significant coral loss, it becomes increasingly important to understand how hurricanes will influence coral skeletal growth parameters on the FKRT.

In the current study, we characterize the impact of Hurricane Irma on the skeletal growth of two abundant and widely distributed Caribbean reef-building corals, *Siderastrea siderea* and *Pseudodiploria strigosa* (Ellis and Solander 1786), on inner and outer reefs along the FKRT. In 2017, Hurricane Irma traversed reef sites where *S. siderea* and *P. strigosa* were previously cored in 2015. This natural phenomenon provided an unprecedented opportunity to assess skeletal growth trends in these coral species, pre- and post-Hurricane Irma, and to characterize any growth recovery. We collected cores of both species and identified annual growth patterns to identify if the hurricane impacted three growth parameters: skeletal density, extension, and calcification. We hypothesize that *S. siderea* and *P. strigosa* corals would exhibit varying responses in each skeletal growth parameter (i.e., increased skeletal density, decreased linear extension, and steady calcification rates) pre- to post-Hurricane Irma. We also hypothesize that the decline in skeletal growth would be more pronounced for conspecifics within inner reef sites (relative to the outer reef) due to lower light availability for autotrophy induced by higher turbidity and lower temperatures due to sea surface heat loss. Alternatively, the corals may revert to heterotrophy and therefore the hurricane would minimally impact all skeletal growth parameters (Hughes and Grottoli 2013).

## Materials and methods

### Site description

**Fig. 1:**
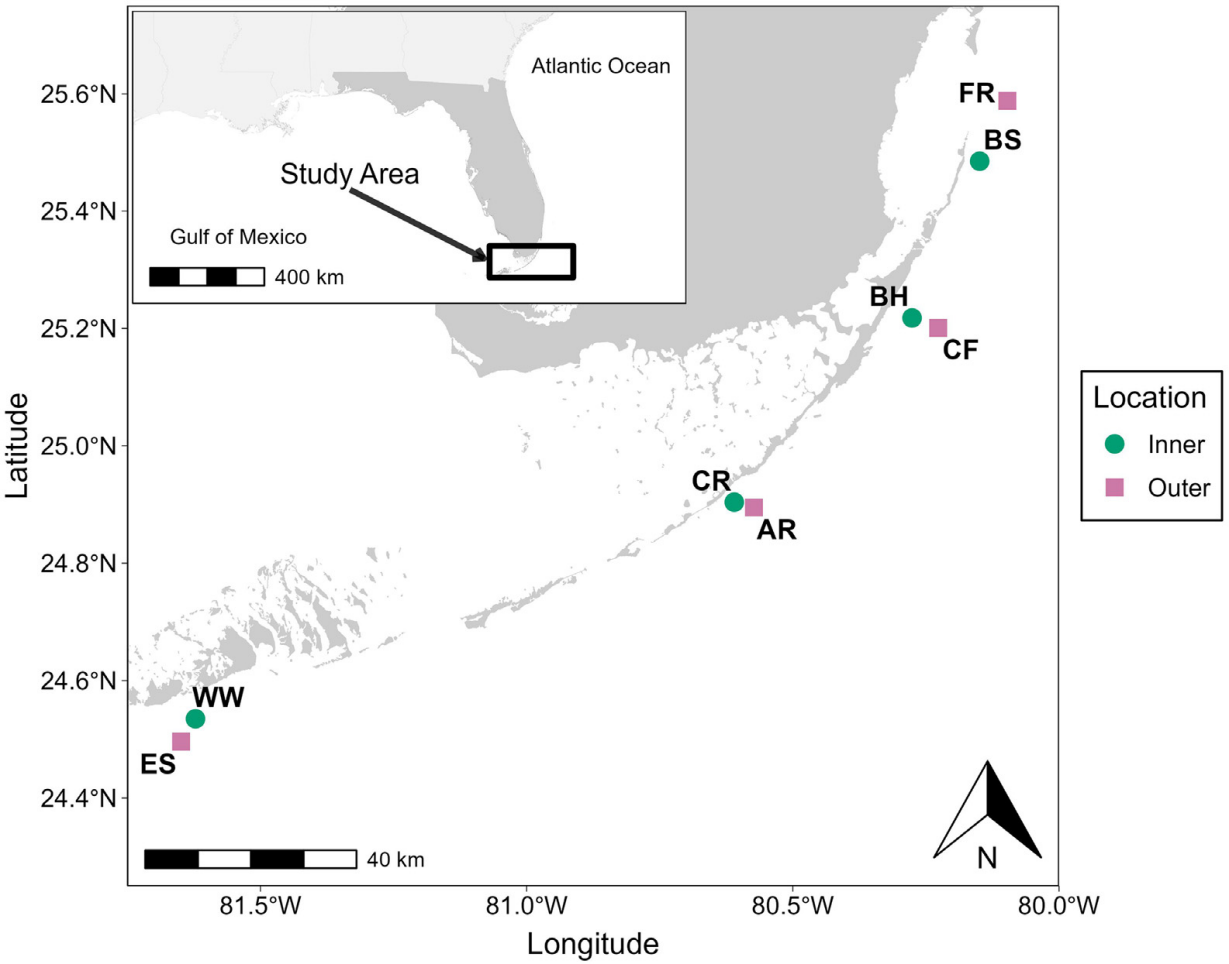
Inner-outer reef micro-core extraction sites along the Florida Keys Reef Tract. Map of core extraction sites where *S. siderea* and *P. strigosa* were obtained. The circles indicate the inner reef sites, while the squares indicate the outer reef sites. Each abbreviation represents the following sites: West Washerwoman (WW) Eastern Sambo (ES), Cheeca Rocks (CR), Alligator Reef (AR), Basin Hills (BH), Carysfort Reef (CF), Bache Shoals (BS), and Fowey Rocks (FR).

Research sites were located along the FKRT. Florida is the only state in the continental United States with extensive shallow tropical coral reef formations near its coasts. This provides 579 km of coral reef inhabited by 45 different species of stony corals, all ranging from 3 to 15 m depth (24° 56′ 37.12“ N 80° 30′ 41.76″ W). We chose *S. siderea* and *P. strigosa*, two commonly found stony corals that inhabit the inner and outer reefs on the FKRT. *Siderastrea siderea* and *P. strigosa* coral micro-cores were obtained from eight sites along the inner and outer reefs of the upper, middle, and lower portions of the FKRT (Fig. 1). The four inner reef sites extending northwards include Washerwoman (WW), Cheeca Rocks (CR), Basin Hill Shoals (BH), and Bache Shoals (BS). The four outer reef sites include Eastern Sambo (ES), Alligator Reef (AR), Carysfort Reef (CF), and Fowey Rocks (FR). Detailed descriptions of these reef sites are in Rippe et al. (2018).

### Coral core extraction

*Siderastrea siderea* and *P. strigosa* coral micro-cores were collected from previously-cored coral colonies (Rippe et al. 2018) at 3 to 8 m depth and were extracted using a Nemo V2 Edition Drill (Nemo Power Tools, Las Vegas, NV, USA) with hollow extension rods and a 5‐cm‐diameter wet diamond core bit (BSP Tools/Xinzhen Industrial Park, Kunshan City, Jiangsu, P.R. China). Since 2015, several of the previously cored coral colonies have been lost to stony coral tissue loss disease (Rippe et al. 2019). Therefore, only 13 micro-cores (8 *S. siderea* and 5 *P. strigosa*) were extracted from previously cored colonies. At each of the eight sites, 20 additional colonies that were not previously cored and appeared healthy were randomly selected for micro-core extraction. All micro-cores were ∼10 cm long, producing ∼20 years of coral growth records. After extraction, a cement plug was inserted in the micro-core extraction site, and Z‐Spar® underwater epoxy (Go2Marine, Bellingham, WA, USA) was used to protect the coral from boring organisms. The cores were stored in capped PVC tubes filled with 100% ethanol and taken to the University of North Carolina at Chapel Hill (UNC), where they were dried until further analysis.

### Coral CT scanning and sclerochronology development

Coral micro-cores were scanned using X‐ray computed tomography (CT) at UNC’s Biomedical Research Imaging Center (BRIC). Micro-cores were scanned following procedures described in Rippe et al. (2018). These samples were derived by using a Siemens Biograph mCT scanner (Clinical Imaging Systems, Jupiter, Florida, USA) set to 120 kV, 250 mAs, and 0.06 mm slice thickness (the appropriate setting to view the samples). The images were then reconstructed in 0.1 mm increments. This was made possible using the scanner's H70h “Very Sharp Spine” window. Micro-cores were scanned with coral standards of known density measurements to convert density from CT Hounsfield units to g/cm^−3^. We assessed the average density of each standard in Hounsfield units using Horos v2.0.2 software. Additionally, for all cores scanned, a standard curve was used (Supplementary Fig. S1). Although 33 micro-cores were collected, one micro-core was severely damaged and therefore was omitted immediately from further processing. An R script, based on Rippe et al. (2018), was used to combine “Region of Interest” (ROIs) addressing deposit tracking in each core and create continuous growth patterns for growth parameters (skeletal density, calcification rate, and linear extension). Further details can be found in the supplementary.

### Statistical analysis

#### Annual coral skeletal growth before and after Hurricane Irma

Measurements of coral skeletal growth parameters (skeletal density, linear extension, and calcification rates) were employed to assess coral growth trends before (2009–2016) and after (2017–2018) Hurricane Irma by species (*S. siderea, P. strigosa*), reef location (inner reef, outer reef), and the combination of species and reef location over time. First, the annual skeletal growth parameters were quantified for each coral core collected over approximately six years. Using this information, mean coral skeletal growth parameters were calculated for each coral species within each site by averaging annual measurements of skeletal density, linear extension, and calcification rates across time, pre- and post-Irma. Next, species, location, and time variability in skeletal density, linear extension, and calcification rates were evaluated via coefficients of variation and then by testing for the significance of correlations between each potentially influential factor. Sixty-four percent (21 of 33) of our initially collected coral cores were retained for further analysis. The remaining cores were not used in the current study due to physical damage or HOROS analysis inconsistencies during assessments (Table 1). To investigate trends in coral skeletal growth parameters before and after Hurricane Irma, by species and by reef location, a three-way ANOVA (analysis of variance) was fitted for each growth parameter, as all assumptions were met. The three-way ANOVA generated seven main effects and interaction terms that were used to assess whether Hurricane Irma impacted *S. siderea* and *P. strigosa* skeletal growth on inner and outer reefs on the FKRT. We performed visual analysis of annual coral skeletal growth before and after Hurricane Irma, calculating 95% confidence intervals for skeletal density, linear extension, and calcification rates to understand their main effects and interaction terms.

**Table 1:** Summary of *S. siderea* and *P. strigosa* micro-cores collected along the Florida Keys Reef Tract

CoreID	Site Name	Reef Location	Core Number
*Siderastrea siderea*			
BH1	Basin Hill Shoals	IN	1
BH2	Basin Hill Shoals	IN	2
CR1	Cheeca Rocks	IN	3
CR2	Cheeca Rocks	IN	4
CR3	Cheeca Rocks	IN	5
BH3	Basin Hill Shoals	IN	6
BS1	Bache Shoals	IN	7
BS3	Bache Shoals	IN	8
AR3	Alligator Reef	OUT	9
CF1	Carysfort	OUT	10
CF2	Carysfort	OUT	11
ES1	E Sambo	OUT	12
ES3	E Sambo	OUT	13
ES4	E Sambo	OUT	14
FR2	Fowey Rocks	OUT	15
*Pseudodiploria strigosa*			
CoreID	Site Name	Reef Location	Core Number
BH4	Basin Hill Shoals	IN	16
WW3	W Washerwoman	IN	17
ES2	E Sambo	OUT	18
ES5	E Sambo	OUT	19
FR1	Fowey Rocks	OUT	20
FR5	Fowey Rocks	OUT	21

Summary of samples taken: *S. siderea* (top) and *P. strigosa* (bottom)

#### Yearly skeletal growth changes before and after Hurricane Irma

Besides our analysis of actual annual skeletal growth parameters, we are also interested in the effect of Hurricane Irma on the “change” in all three growth parameters from year to year. Each parameter’s yearly change in skeletal growth was calculated by comparing each annual skeletal growth to the corresponding year. Here, we describe the procedure to generate the year changes using annual skeletal density since the annual linear extension and calcification rates are calculated analogously. For any given coral *i*, and year *t*, between 2012 and 2018, we represent the density of coral *i* in the year *t* as:


(1) \begin{eqnarray*}
\gamma _{i,t}^{\left( D \right)}.
\end{eqnarray*}

To represent the coral density before year *t*, we use the average of the three previous years, given by


(2) \begin{eqnarray*}
\bar{\gamma }_{i,t - }^{\left( {\mathrm{D}} \right)}{\mathrm{ = }}{{1} \!\mathord{\left/ {\vphantom {1 3}}\right.} \!{3}}\left( {\gamma _{i,{\mathrm{t}} - 3}^{\left( {\mathrm{D}} \right)} + \gamma _{i,t - 2}^{\left( {\mathrm{D}} \right)} + \gamma _{i,t - 1}^{\left( {\mathrm{D}} \right)}} \right)
\end{eqnarray*}

From this, we define the change in density for year *t* as


(3) \begin{eqnarray*}
\Delta _{i,t}^{\left( D \right)} = \gamma _{i,t}^{\left( D \right)} - \bar{\gamma }_{i,t - }^{ - \left( D \right)}
\end{eqnarray*}

Analysis of the “yearly growth changes” also allows us to address other issues that are not accounted for in the analysis of the original annual skeletal growth parameters. Annual skeletal growth is not fully independent (i.e., they were derived from a set of 21 cores), and thus growth values from a single coral over time are correlated. Analysis of yearly changes in coral growth allows us to address the random effect of individual corals on the model. A three-way ANOVA was fitted for each yearly change in coral annual skeletal growth for each parameter.

A multiple testing adjustment was also run against each statistically significant interaction found by the three-way ANOVA to verify significance further.

## Results

### Coral annual skeletal growth before and after Hurricane Irma

**Fig. 2:**
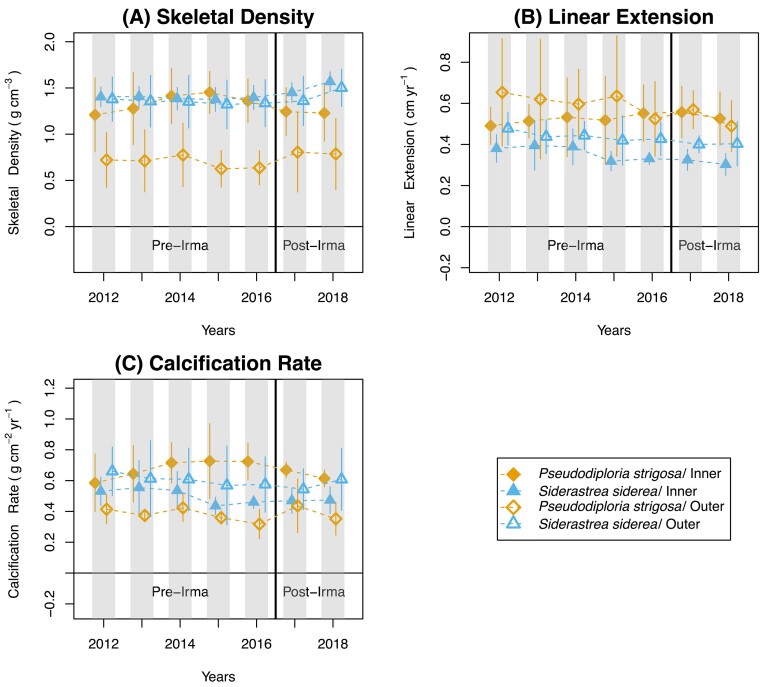
(A–C) Coral annual skeletal growth parameters across reef locations. Annual growth data, skeletal density (A), linear extension (B), and calcification rate (C), are shown for *Siderastrea siderea* and *Pseudodiploria strigosa* pre-Hurricane Irma (2009–2016) and post-Hurricane Irma (2017–2018). Each color is coded by species and location combination. *Siderastrea siderea*/inner (filled triangle) *S. siderea*/outer (open triangle) *P. strigosa*/inner (filled diamond); *P. strigosa*/outer (open diamond). Dashed lines show average trends. The solid black line indicates the year Hurricane Irma transversed in 2017.

Annual skeletal growth parameters (skeletal density, linear extension, and calcification rates) were not significantly different before and after Hurricane Irma (Fig. 2A–C; Supplementary Figs. S2 and S3, Supplementary Table S1; *P* > 0.05). Furthermore, Hurricane Irma had no effect on coral annual skeletal growth parameters by species (*S. siderea, P. strigosa*), location (inner reef; outer reef), or their interaction (Supplementary Table S1; *P* > 0.05). Even though not significant, results suggest an upward trend toward higher annual skeletal density for *S. siderea* pre- to post-Irma, regardless of reef location (Fig. 2A). This upward trend was not observed for *S. siderea* linear extension and calcification rates (Figs. 2B and C). Similar trends were not observed for all growth parameters in *P. strigosa*, regardless of reef location (Fig. 2A–C) species, location, and the interaction of species and location were found to be significant (Supplementary Table S1; *P* < 0.05), and some trends, though not significant, were seen within at least one annual coral skeletal growth parameter. To explore these relationships further, we conducted a visual analysis in Supplementary Fig. S4A–D to gain better insight into their effects on coral annual growth parameters. Again, the visual analysis (overlapping bars) reconfirms that coral annual skeletal density was not significantly different before and after Hurricane Irma. Further details can be found in the supplementary.

### Yearly changes in annual coral skeletal growth before and after Hurricane Irma

**Fig. 3:**
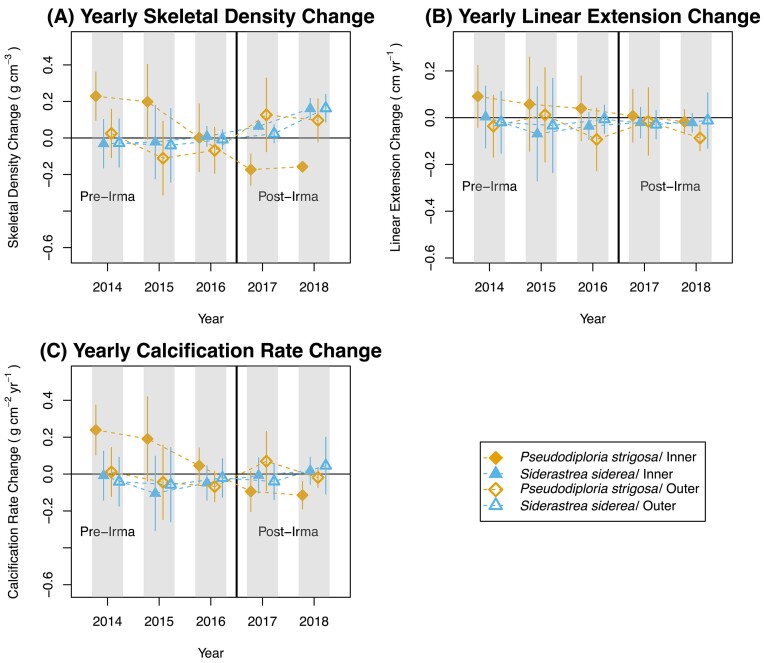
(A–C) “Yearly” Changes in Coral Annual Skeletal Growth Parameters. Some yearly skeletal growth parameters are potentially impacted by hurricane exposure. Yearly skeletal growth parameter changes, skeletal density (A), linear extension (B), and calcification rate (C) are shown for *Siderastrea siderea* and *Pseudodiploria strigosa* pre-Hurricane Irma (2009–2016) and post-Hurricane Irma (2017–2018). Each color is coded by species and location combination. *S. siderea*/inner (filled triangle); *S. siderea*/outer (open triangle); *P. strigosa*/inner (filled diamond); *P. strigosa*/outer (open diamond). Dashed lines show average trends. The solid black line indicates the year Hurricane Irma transversed in 2017.

In addition to the analysis of the direct skeletal growth measurements, we are also interested in whether Irma had an effect on the change in all three growth parameters from one year to another (i.e., yearly changes in annual skeletal growth). Our results reveal yearly changes in annual skeletal density varied before and after Hurricane Irma (Fig. 3A-C; Supplementary Table S2; *P* < 0.0005). However, yearly changes in annual skeletal extension and yearly changes in annual calcification rates were not significant (Fig. 3B and C; Supplementary Table S2; *P* > 0.05). Because the yearly changes in skeletal density were also significant for the interaction of Irma by species, location, and by location and species (*P* < 0.005), we use visual analysis to understand better the nature of the yearly changes in skeletal density.

### Visual analysis of the yearly change in annual coral skeletal growth before and after Hurricane Irma

**Fig. 4:**
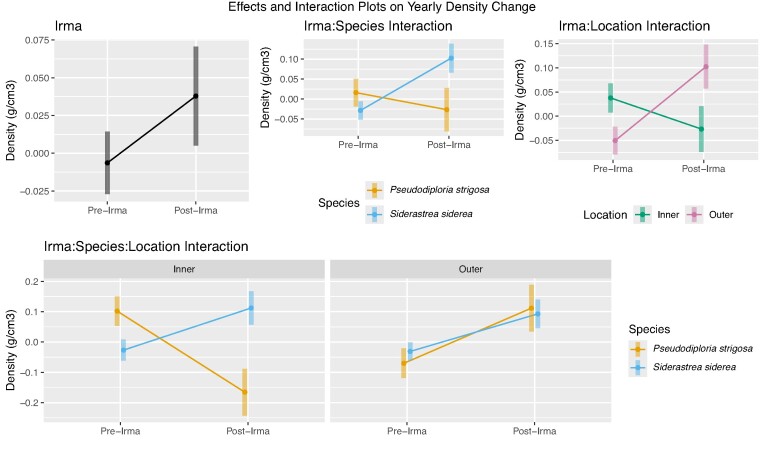
(A–D) Visualization of the “Yearly” changes in coral average annual skeletal density across reef locations. Yearly skeletal density is potentially impacted by hurricane exposure. Visualizations of the average yearly skeletal density growth change are given for time (A), species (B), location (C), and species and location (D). Species (*S. siderea* and *P. strigosa* )and locations (inner and outer) are listed. .

Mean values of yearly changes are shown with 95% confidence intervals for each parameter. The visual analysis (non-overlapping bars) reconfirms that the yearly change in coral annual skeletal density was significantly different before and after Hurricane Irma, with coral skeletal density increasing pre- to post-Hurricane Irma (Fig. 4A). Yearly change in annual skeletal density decreased for *P. strigosa* after exposure to Hurricane Irma but increased for *S. siderea* (Fig. 4B). Furthermore, yearly change in annual skeletal density decreased in the inner reef post-exposure to Hurricane Irma, while values increased in the outer reef increased (Fig. 4C). Within the inner reef, the yearly change in annual skeletal density for *P. strigosa* decreased after exposure to Hurricane Irma, while values for *S. siderea* increased. In the outer reefs, both species exhibited increased yearly change in annual skeletal density (Fig. 4D).

## Discussion

### Hurricane Irma effects on coral annual skeletal growth

Hurricane Irma did not affect coral annual skeletal growth across the FKRT when comparing values for the three annual growth parameters (i.e., skeletal density, linear extension, and calcification rates) before and after the storm. However, upward trends suggest shifts in annual skeletal density for *S. siderea* in both locations.

The main reason is that the coupled skeletal growth bands of the two slow-growing mounding corals, *S. siderea* and *P. strigosa*, investigated in the current study were unaffected by the storm. On the FKRT and other regions in the Caribbean, *S. siderea* and other mounding corals deposit a low-density growth band from December to May and a high-density growth band from June to November (Guzman and Tudhope 1998; Castillo et al. 2012; Rippe et al. 2018). Thus, if neither of these sub-annual growth bands were not impacted by the storm, no growth effects would be detected in the annual skeletal growth parameters. It is also possible that Hurricane Irma negatively impacted one of the two growth bands (i.e., the high-density growth band deposited from June to November, after the storm passed in September), and the negative effect was diminished by the subsequently deposited growth band.

In the current study, the skeletal growth analysis was conducted at the annual level, thus any sub-annual variation in growth would not be detected. It should also be noted that hurricanes and other storms are known to have a more significant impact on the growth of branching corals that are more directly impacted by increased wave activity and turbulence generated by these storms (Highsmith et al. 1980; Mumby et al. 2011; Rogers 1993; Viehman et al. 2020). However, the growth impacts on mounding species like those invested here are often more indirect and less pronounced (Peckol et al. 2003; Pascoe et al. 2021) and are often tied to longer-term changes generated by the storm such as increased sedimentation, decreased light availability, and other related storm-generated factors (Cheriton et al. 2019; Edmunds et al. 2019; Takesue et al. 2021).

Another possible explanation for the lack of an effect on coral annual skeletal growth by Hurricane Irma is that the corals sampled on the FKRT during the present study are those that have survived years of chronic stress. This would include impacts such as warming, acidification, and a variety of local anthropogenic stressors (Hughes et al. 2003; Manzello et al. 2012; Kuffner et al. 2015; Manzello 2015) and various acute stressors such as coral bleaching, other storms, and cold snaps (Burns 1985; Kemp et al. 2011; Gintert et al. 2018;; Wilkinson and Souter 2008). Further explanation can be found in the supplementary. Although not significant, our results suggest an upward trend towards higher annual skeletal density for *S. siderea*, regardless of reef location (Fig. 2A). *Siderastrea siderea* is known to be a relatively resilient and adaptable stony coral species (Kemp et al. 2011; Castillo et al. 2014; Davies et al. 2018), but due to its slow-growing nature (Maupin et al. 2008), greater average skeletal density is common throughout the FKRT (Rippe et al. 2018). The upward trend in skeletal density observed for *S. siderea* here is likely driven by the decreased environmental conditions (i.e., increased sedimentation and decreased light availability) generated by the storm. Decreased light availability suppressed light-enhanced calcification, causing the coral to grow at a much slower rate (Bosscher 1993; Mallon et al. 2022). This means that the calcium carbonate deposition decreases causing the skeletal material deposited to be denser. Indeed, Guzman and Tudhope (1998) chose *S. siderea* for their study due to its durability. However, it also found that although it may be durable and survive acute disturbances, it still exhibits evidence of substantial environmental changes in its skeletal structure. It is unclear why a similar upward trend in skeletal density was not observed for *P. strigosa* after the storm. Like *S. siderea, P. strigosa* is also a relatively resilient coral species. Viehman et al. (2020) found that although *P. strigosa* found along the Caribbean post-2017 hurricane exposure had a high number of damaged colonies, it had a very low frequency, 3–9%, of damaged corals. As the frequency is low, the skeletal density may not be as impacted in this species. We also may not see a higher annual skeletal density for *P. strigosa* because the cores sampled were not from a colony that was negatively impacted by storm exposure.

### Coral skeletal growth varied by species and location

Although Hurricane Irma had no effect on coral annual skeletal growth parameters, we found that all three growth parameters exhibited some varying trends by species, location, and their interaction with the FKRT. First, inner reef corals exhibited higher annual skeletal density than their outer reef counterparts for both species. Inner reef systems are susceptible to introductions of runoff, suspended sediment, lower light, and lower temperatures, all of which are known to impede coral growth and thus increase coral annual skeletal density (Rippe et al. 2018). Outer reefs usually have clearer water due to their lack of immediate impact from runoff or land-based coastal influences and can sustain higher calcification rates. Because the location changes coral exposure levels, coral growth parameters change along inshore-offshore transects (Hughes 1987; Lough and Barnes 1992). On the FKRT, inner reef systems have lower temperatures and higher variability in temperature (Kemp et al. 2011), suggesting the possibility of slowed overall growth and higher annual density in inner reefs than outer reefs.

Second, *S. siderea* exhibited higher annual skeletal density than *P. strigosa*. Corals have been adaptable enough to environmental changes to sustain some skeletal growth parameters, such as linear extension and calcification rates, but not shifts in skeletal density. As previously stated, the average skeletal density of *S. siderea* is greater throughout the FKRT, while linear extension and calcification were comparatively constant (Rippe et al. 2018). With fairly steady calcification rates, high density indicated slower growth among *S. siderea* (Mallon et al. 2022). Gender can also influence density deposit despite location (Tortolero-Langarica et al. 2016). It is possible that the gender of the *S. siderea* corals from which the micro-cores in the current study were collected could have caused the observed density differences. Female *S. siderea* have thinner tissue and a denser skeleton, and although there are no gender-related differences in calcification rate, skeletal density may differ (Carricart-Ganivet et al. 2013; Benson et al. 2019). Although multiple samples of *S. siderea* and *P. strigosa* were collected and every effort was made to sample across the reef and at each site randomly, it is possible that the majority of our *S. siderea* coral were female. This could explain the density difference observed between the two species in the current study.

Third, *S. siderea* exhibited higher annual skeletal density than *P. strigosa* in inner and outer reefs. Again, temperature could also drive this effect where inner reef temperatures are cooler. *Siderastrea siderea* in the Florida Keys was minimally impacted by cooler water environments compared to other species (Kemp et al. 2011), which could explain a slightly higher average skeletal density than *P. strigosa* in the inner reefs. Castillo et al. (2011) found declining skeletal extension in *S. siderea* for forereefs in Belize. A sustained or decreased linear extension is often coupled with increased skeletal density. This might explain the small difference in *S. siderea* average skeletal densities between each location, yet the very large difference in *S. siderea* average skeletal density compared to *P. strigosa* in the outer reef.

Fourth, we also observed that *P. strigosa* exhibited higher annual skeletal density in inner reefs than in outer reefs. One explanation for this is that location can support feeding shifts due to environmental differences, similar to stress-inducing events (Houlbrèque et al. 2003). Corals are able to shift their feeding habits based on their exposure to their environment, which can vary in light availability, nutrient availability, and temperature (Houlbrèque and Ferrier-Pagès 2009; Ezzat et al. 2016). When in extremely stressful environments, corals may bleach, expelling algae used for photoautotrophy but shift feeding strategies to heterotrophy to sustain endosymbiotic algae, coral host, and skeletal growth (Hughes and Grottoli 2013). Outer reefs are known to have higher temperatures but clearer waters. Inner reef systems are known to have higher sediment and lower light availability. These location differences allow for different growth strategies within the coral to survive. Outer reefs are known to conduct photosynthesis and produce lower δ^15^N levels due to high light availability. This feeding strategy can shift to a higher δ^15^N once the species is introduced to a low-light environment (Baumann et al. 2021). This might be why our findings support location differentiation in growth among inner and outer reef *P. strigosa* corals.

### Hurricane Irma effect on yearly change in annual skeletal density

Because trends were higher in annual skeletal density for *S. siderea* regardless of reef location, we decided to assess the impact of Hurricane Irma on yearly annual skeletal growth change. This assessment includes taking the average of the three previous years and comparing it to the year in question. The results were then grouped by pre- and post-Hurricane Irma exposure, thus allowing us to see average annual growth change before and after Hurricane Irma transverse the FKRT.

The yearly change in annual skeletal density increased after exposure to Hurricane Irma. This change was not observed for the yearly change in annual linear extension and the yearly change in annual calcification rates (Fig. 3B and C). The increased yearly change in annual skeletal density observed in the current study could be driven by the exposure of the corals to environmental shifts induced by the hurricane compared to the previous years of growth. Temperature fluctuations and light availability are most commonly listed as influencing coral skeletal density based on the cyclical variations in weather patterns characteristic of each season (Risk and Sammarco 1991). Guzman and Tudhope (1998) found that coral skeletal density increased in corals from cooler to warmer seasonal cycles. Similarly, coral skeletal density increased from the heavy rainy season, inducing low light levels on reefs to the dryer summer intervals resulting in increased visibility and higher irradiance of the reef. As previously stated, hurricanes induce cooling effects, decrease light availability, and increase runoff (Edmunds et al. 2019). Takesue et al. (2021) found that hurricanes also cause structural damage to coastlines, increasing the amount of introduced sedimentation during and after a storm. As prolonged rainfall from this ocean evaporative cooling can increase sediment and runoff introduction (Trenberth et al. 2018), the structural integrity of the region can sustain high sediment impact. If enough physical damage and sediment are introduced to the reef, this would cause a lasting impact of decreased light availability, high turbidity, and runoff, potentially seen in the yearly average and not the annual values.

Yearly changes in annual skeletal density decreased in the inner reefs post-exposure to Hurricane Irma, while values increased in the outer reef. Risk and Sammarco (1991) found that the skeletal density of *Porites* increased significantly in the Great Barrier Reef with an increased distance from inshore reefs to offshore reefs. Again, this dynamic was coupled with the increased introduction of nutrients and compromised light availability inshore. In 2017, Hurricane Irma disrupted the availability of underwater light at the surface, maximum daily light, and integrated levels. This was all driven by heavy rainfall and sedimentation (Edmunds et al. 2019). This could have created a large enough disturbance in the inner reef systems to exacerbate pre-existing conditions and shift the average skeletal density levels but sustain overall calcification rates. Unlike annual growth, yearly growth changes showed an average skeletal density change increase, which may be caused by sustained environmental shifts, enough stress, or a lack of ability to withstand more.

*Siderastrea siderea* exhibited increased yearly change in annual skeletal density post-Hurricane Irma, while the yearly change in annual skeletal density decreased for *P. strigosa*. Over the past 8 years, there was a significant decline in the skeletal density of *P. strigosa*, which could be identified as a long-term trend, specifically in the Florida Keys (Rippe et al. 2018; Manzello et al. 2021). With the introduction of Hurricane Irma, the coral may have adapted to environmental changes and the hurricane may not have caused enough change or exacerbated existing conditions that the coral was already experiencing. However, in the case of *S. siderea*, continued disturbance exposure in the FKRT increased coral calcification capacity (CCC) but decreased in other regions. This variability in CCC depends on stress exposure and aids in the decline of coral density deposit, cover, and overall modification of benthic community composition (Courtney et al. 2020). With continuous exposure to disturbances, *S. siderea* can lose some of its stress tolerance and increase in density due to its lowered resilience to another disturbance.

*Pseudodiploria strigosa* exhibited a decrease in the yearly change in skeletal density in the inner reefs post-Hurricane Irma. Furthermore, the changes in yearly skeletal density increased for both *S. siderea* and *P. strigosa* corals in the outer reef. This could be supported by density increasing with distance from shore (Risk and Sammarco 1991) as well as exacerbated environmental characteristics of the location during and after a storm. For example, hurricanes influence coral growth by introducing sediment and turbidity (Crabbe 2009). Hurricane Maria introduced sediments and runoff to reef systems in Puerto Rico, but in the 8 months following, hurricane-related alterations of landscapes influenced runoff patterns, and fine sediment was found in distant outer reefs with the help of the region’s storm exposure and hydrodynamics (Cheriton et al. 2019; Takesue et al. 2021). Outer reef systems deemed delicate, such as in Mexico, saw drastic decreases in coral cover after exposure to Hurricane Gilbert (Fenner 1991). In the FKRT, Somerfield et al. (2008) found that after exposure to multiple acute disturbances, bleaching events in 1997 and 1998, and exposure to Hurricane George resulted in no to low recovery of *Scleractinia and Milleporina* in offshore reefs, suggesting that they were no longer resilient. The outer reefs are known to be delicate, so storm alterations would weaken reef resilience, instill a lasting impact that would reach outer reef habitats, and influence yearly changes in the skeletal growth of both *S. siderea* and *P. strigosa*. Inner reefs, however, usually susceptible to variable conditions, could exacerbate stress, increase yearly skeletal density change in S. *siderea*, and improve adaptability in *P. strigosa*, therefore decreasing yearly skeletal density change after exposure to Hurricane Irma.

## Conclusion

The current study’s findings suggest that hurricanes may not impact coral annual skeletal growth, at least over the intervals before and after the storm assessed here. However, we observed increased yearly change in annual skeletal density after the storm, driven primarily by density differences in *S. siderea*. As hurricanes continue to increase in frequency and strength, this increased skeletal density effect could become more prevalent across reef ecosystems worldwide. Hurricane Irma did not affect the annual skeletal growth of corals on the FKRT. Although not significant, our results also suggest an upward trend towards higher annual skeletal density for *S. siderea*, regardless of reef location. This supports our findings that species and location influence some skeletal growth parameters. This interaction was seen in inner reefs, as corals exhibited higher annual skeletal density than their outer reef counterparts. *Siderastrea siderea* also maintained high skeletal density compared to *P. strigosa* in both reefs. *Pseudodiploria strigosa* skeletal density was higher in inner reefs than in outer reefs. Yearly skeletal density change is impacted by Hurricane Irma potentially from environmental changes induced by the storm. This was also influenced by species interaction when combined with Hurricane Irma exposure but only for skeletal density changes. Outer reefs increase yearly skeletal density change and inner decrease post-Hurricane Irma exposure. *S. siderea* increased in yearly skeletal density change post-Hurricane Irma exposure, unlike *P. strigosa*, which decreased. *Pseudodiploria strigosa* inner reef coral decreased yearly skeletal density change post-Hurricane Irma exposure, but *S. siderea* increased. Both species in the outer reef systems showed an increase in yearly skeletal density change.

Overall, our results reveal that some corals on the FKRT exhibit higher yearly skeletal density change deposits after storm exposure but sustain overall linear extension and calcification rates. Because of the relationship between these skeletal growth parameters, high density is expected to lead to a decrease in linear extension and then a decrease in calcification rates.

There are several limitations that should be considered when interpreting the results of our study. First, in the current study, 33 cores were originally collected, but due to physical damage to coral and some difficulties in the processing of some cores using HOROS, our sample size was reduced to 21. Second, we cannot make causal conclusions on Hurricane Irma’s impacts on skeletal growth since there was no control reef group (i.e., where the same two species of coral within the same region were not exposed to Irma). However, we can say yearly skeletal growth change shifts were observed in corals that were exposed to Hurricane Irma. Third, addressing longer temporal intervals before and after the storm would have given us more robust averages and data.

Future research addressing this or a similar question on the impact of tropical storms on coral growth should include: (1) a larger sample size; (2) a control set of cores from outside of the hurricane’s path; (3) longer temporal interval than 10 years of growth prior and after the storm and address all storm passes within that time frame; and (4) assessment of coral-coupled growth bands (i.e., high- and low-density bands) deposited seasonally since tracking bi-annual growth patterns could also uncover a deeper understanding of how extensive hurricanes are impacting coral growth.

## Author contributions

A.G. and K.C. conceptualized the study, acquired all funding and resources, and carried out all fieldwork with volunteer divers. A.G. conceptualized statistics, performed all scanning methods, and all project administration responsibilities. J.S. performed ANOVA and all formal analyses and corresponding Figs. Writing was led by A.G., with all co-author input in reviewing and editing.

## Supplementary Material

icae128_Supplemental_File

## Data Availability

The data underlying this article will be shared on reasonable request to the corresponding author.
