# Supplementary material for: Hurricane Irma Linked to Coral Skeletal Density Shifts on the Florida Keys Reef Tract
Source: Integr Comp Biol. 2024 Aug 5;64(4):1064–77. doi: 10.1093/icb/icae128 (PMC11518571; doi:10.1093/icb/icae128)
Supplement: icae128_Supplemental_File [file icae128_supplemental_file.docx]

**APPENDIX:  SUPPLEMENTAL DESCRIPTIONS, DATA, FIGURES, TABLES**

**MATERIALS AND METHODS**

**Coral CT Scanning**

Horos was also used to measure linear extension by coupling the width of each annual density band and calcification, or the combination of density and linear extension. Using Horos, each file was altered into three‐dimensional medical images. To assess differentiating high- and low-density growth bands, a “Mean” standard projection 10‐mm thick was placed at the center of each core as a rectangular prism. After scanning through the 3-D scan, the best image and resolution was saved for further processing. All obtained images were downloaded as individual DICOM files.

**Sclerochronology Development**

Coral high- and low-density annual band couplets were manually delineated. This delineation started from the top of the micro-core, where the most recent band was deposited down the length of the core to establish a chronological standard. A set of three linear transects were also drawn through the length of each core image using the Horos tool “Region of Interest” (ROI). The ROIs were used to standardize deposit quantifications to avoid inconsistent deposit tracking in the core. Linear transects were delineated to avoid bioerosion or discontinued growth along the core. Some cores had one transect with paired short ROIs. This technique was used in the presence of non-linear growth axes to reach the full length of the core. Additional details of the sclerochronology development procedures are described in [(Rippe et al., 2018)](https://www.zotero.org/google-docs/?broken=grGawh).

**RESULTS**

**Visual Analysis of Annual Coral Skeletal Growth before and after Hurricane Irma**

As observed in Figure 3A-D, each visualized interaction was based on an average of each growth parameter set at 95% confidence intervals. Again, the visual analysis (overlapping bars) reconfirms that coral annual skeletal density was not significantly different before and after Hurricane Irma (Figure 3A). In general, annual skeletal density was significantly higher in the inner reefs (1.36 g cm^3^) compared to the outer reefs (1.06 g cm^3^)  (Figure 3B). Additionally, *P. strigosa* annual skeletal density was much lower (1.01g cm^3^) than that of *S. siderea* (1.42 g cm^3^) (Figure 3C). However,  *S. siderea* annual skeletal density was similar regardless of reef location, while *P. strigosa* annual skeletal density was higher in the inner reef (Figure 3D).

**DISCUSSION**

**Hurricane Irma Effects on Coral Annual Skeletal Growth (continued)**

These remaining colonies are likely more resistant or resilient to subsequent acute stressors such as those induced by Hurricane Irma in September 2017. Indeed, Lirman & Fong (1997) found that on the FKRT, the disturbance history of a reef can influence the outcome of new disturbances and that periodic passage of storms can increase the resilience of surviving colonies. This could possibly explain why no effects of Hurricane Irma on annual coral growth were detected in the current study. In contrast, on the Belize Barrier Reef System (BBRS), [Carilli et al. (2009)](https://www.zotero.org/google-docs/?broken=VgfCNO) found that coral growth rates at reef sites that experience higher local anthropogenic stressors were more impacted by an acute stressor (in their case, a major bleaching event) than growth rates at sites historically exposed to lower stress. The authors concluded that exposure to chronic local stressors reduces coral resilience to new acute stressor(s).  However, similar to the results of the current study, Carilli et al. (2009) found that *Montastraea faveolata,* (Ellis and Solander, 1786) from highly stressed reefs on the BBRS, exhibited no growth effects from Hurricane Mitch, a category 5 storm that struck the region in 1998. Additionally, the authors found very little evidence of any previous storm impacts. They hypothesize that a larger acute disturbance, such as the 1998 mass bleaching event in the region (Aronson et al., 2000), may have overshadowed the immediate impacts of other acute disturbances, such as hurricanes. Similarly, on the FKRT,  growth patterns of coral exposed to a higher impacting acute disturbance may be overshadowing previous hurricane exposures.

**
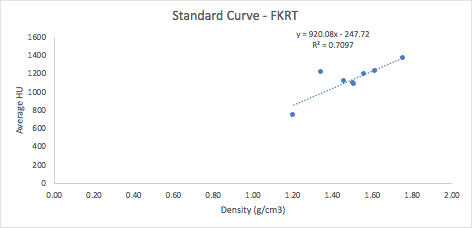
Figure S1 Calibration curve for Density.** Density was achieved by converting CT Hounsfield units based on known densities of nine coral standards.

**
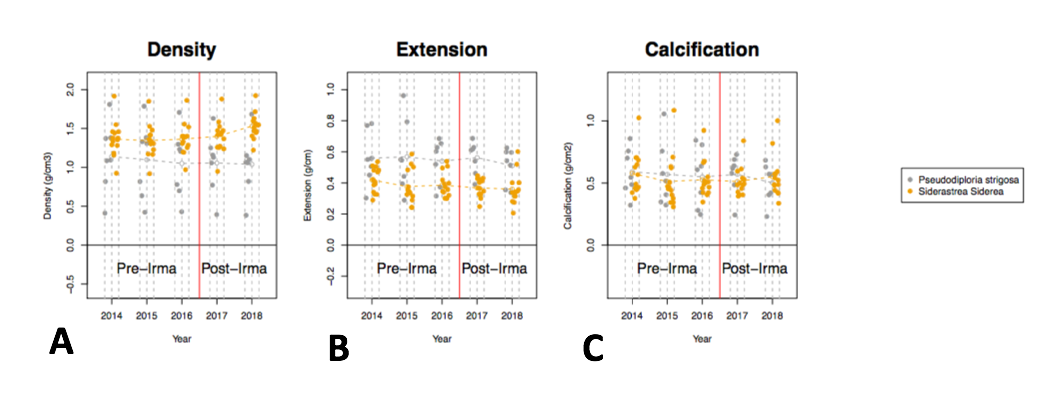
**

**Figure S2A-C Trends are shown toward a shift in *Siderastrea siderea* annual skeletal density.** Annual growth data, skeletal density (A) linear extension (B) and calcification rate (C), of *Siderastrea siderea* and *Pseudodiploria strigosa* pre–Hurricane Irma (2012-2016) and post Hurricane Irma (2017-2018). Each color is coded by species *S. siderea* (orange) and *P. strigosa* (gray). Dashed lines show average trends and match the color scheme of the interaction listed above. The red line indicates the year Hurricane Irma transversed in 2017.

**
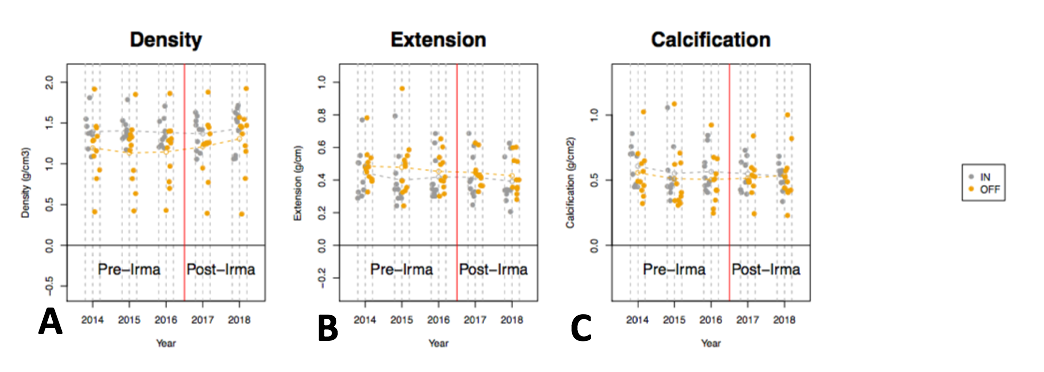
**

**Figure S3A-C Trends are shown toward a shift in annual skeletal density in both reefs.** Annual growth data, skeletal density (A) linear extension (B) and calcification rate(C), of inner and outer reef systems pre–Hurricane Irma (2012-2016) and post Hurricane Irma (2017-2018). Each color is coded by location inner reef (gray) and Outer reef (orange). Dashed lines show average trends and match the color scheme of the interaction listed above. The red line indicates the year Hurricane Irma transversed in 2017.

**
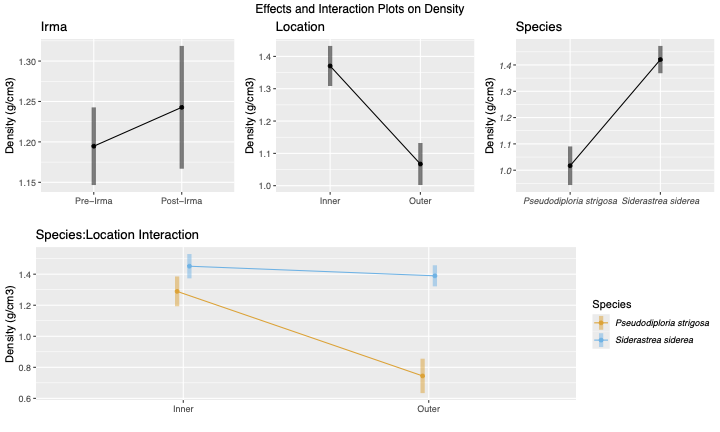
Figure S4A-D: Visualization of Differences in Coral Annual Skeletal Density Across Reef Locations.** Species and location impact skeletal density parameters. Visualization of annual skeletal density growth average comparing (A) time: pre- and post-Hurricane Irma impacts, (B) species: *S. siderea* (blue) and *P. strigosa* (orange), (C) location: inner reefs and outer reefs and (D) species/location interaction. All were fit to have error bars with 95% confidence intervals.

**
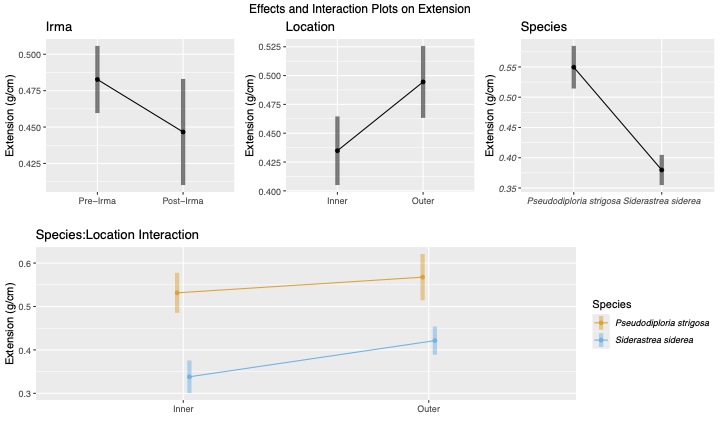
**

**Figure S5A-D Annual skeletal linear extension growth averages are not impacted by Hurricane exposure.** Visualization of the average annual skeletal linear extension growth interactions are shown (A) pre–Hurricane Irma and post Hurricane Irma (B) in inner and outer reefs (C) based on species (*Siderastrea siderea* and *Pseudodiploria strigosa*) (D) and species/location interaction with *Siderastrea siderea* (blue) and *Pseudodiploria strigosa* (orange) in inner (left) and outer reefs (right) pre- and post-Hurricane Irma exposure.

**
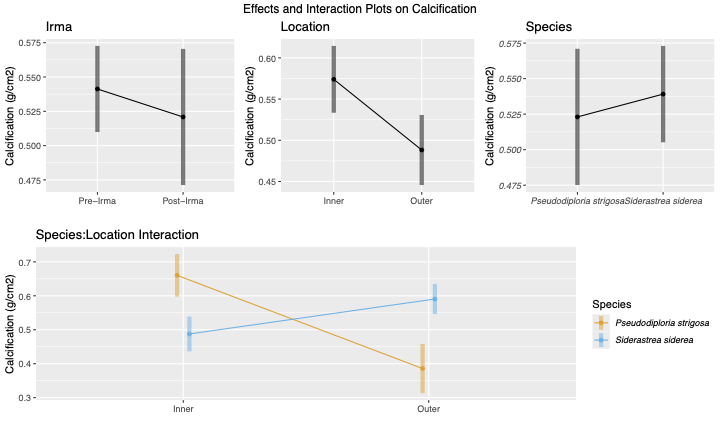
**

**Figure S6A-D Annual skeletal calcification rate averages are not impacted by Hurricane exposure.** Visualization of the average annual skeletal calcification rate interactions are shown (A) pre–Hurricane Irma and post-Hurricane Irma (B) in inner and outer reefs (C) based on species (*Siderastrea siderea* and *Pseudodiploria strigosa*) (D) and species/location interaction with *Siderastrea siderea* (blue) and *Pseudodiploria strigosa* (orange) in inner (left) and outer reefs (right) pre- and post-Hurricane Irma exposure.

**
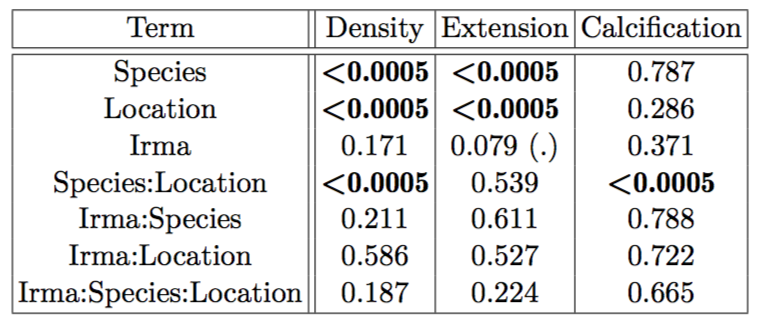
**

**Table S1 All Hurricane Irma interactions do not impact annual skeletal density growth.**

Summary of 3-way ANOVA results on all growth factors (species, location, and Hurricane Irma) to assess impacts on annual skeletal growth parameters (skeletal density, linear extension, and calcification rate).


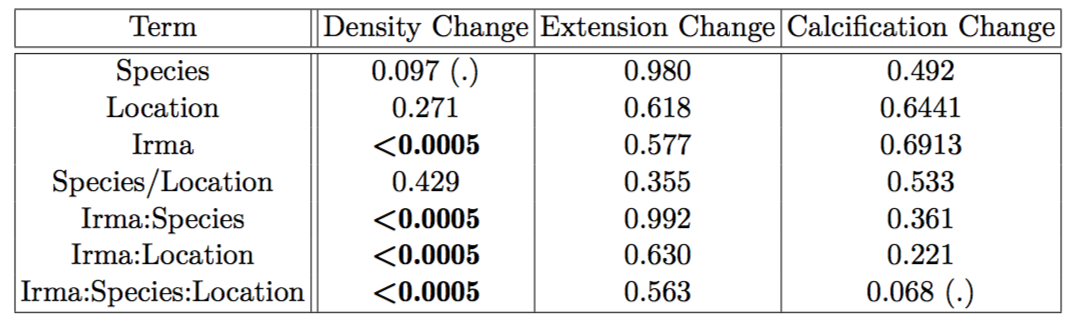


**Table S2 All Hurricane Irma interactions potentially impact yearly skeletal density change.** Summary of 3-way ANOVA results on all growth factors (species, location, and Hurricane Irma) to assess impacts on yearly skeletal growth parameter change (skeletal density, linear extension, and calcification rate.
